# Supplementary material for: A Humanized Yeast Phenomic Model of Deoxycytidine Kinase to Predict Genetic Buffering of Nucleoside Analog Cytotoxicity
Source: Genes (Basel). 2019 Sep 30;10(10):770. doi: 10.3390/genes10100770 (PMC6826991; doi:10.3390/genes10100770)
Supplement: Supplementary file 1 [file genes-10-00770-s001.zip › Additional_Files/Additional_File7_GOTermHeatmaps.zip!/Additional_File7_GOTermHeatmaps/DNA_damage_checkpoint.pdf]

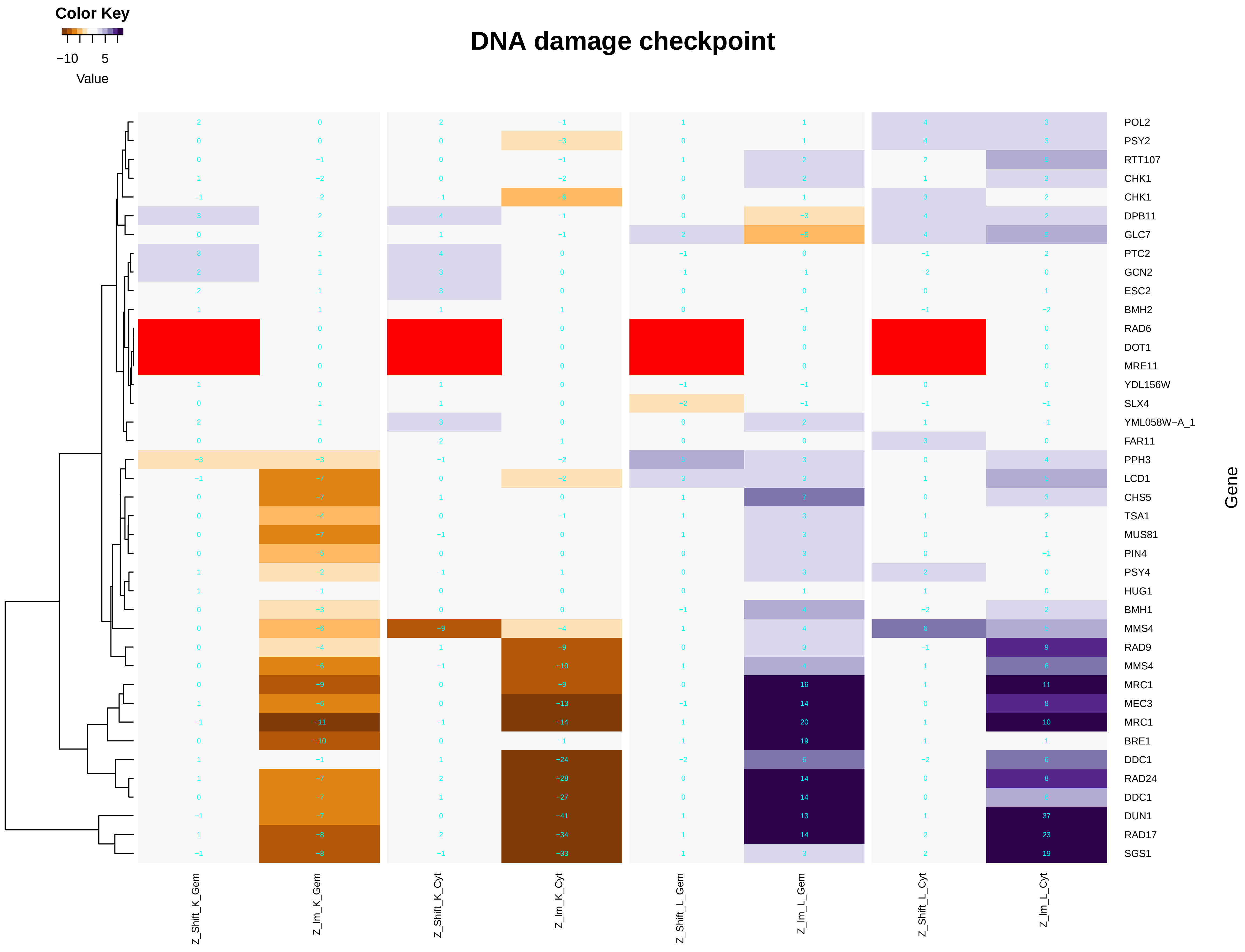

DNA damage checkpoint
Color Key
−10 5
Value
POL2
PSY2
RTT107
CHK1
CHK1
DPB11
GLC7
PTC2
GCN2
ESC2
BMH2
RAD6
DOT1
MRE11
YDL156W
SLX4
YML058W−A_1
FAR11
PPH3
LCD1
CHS5
TSA1
MUS81
PIN4
PSY4
HUG1
BMH1
MMS4
RAD9
MMS4
MRC1
MEC3
MRC1
BRE1
DDC1
RAD24
DDC1
DUN1
RAD17
SGS1
Gene
Z_Shift_K_Gem
Z_Im_K_Gem
Z_Shift_K_Cyt
Z_Im_K_Cyt
Z_Shift_L_Gem
Z_Im_L_Gem
Z_Shift_L_Cyt
Z_Im_L_Cyt

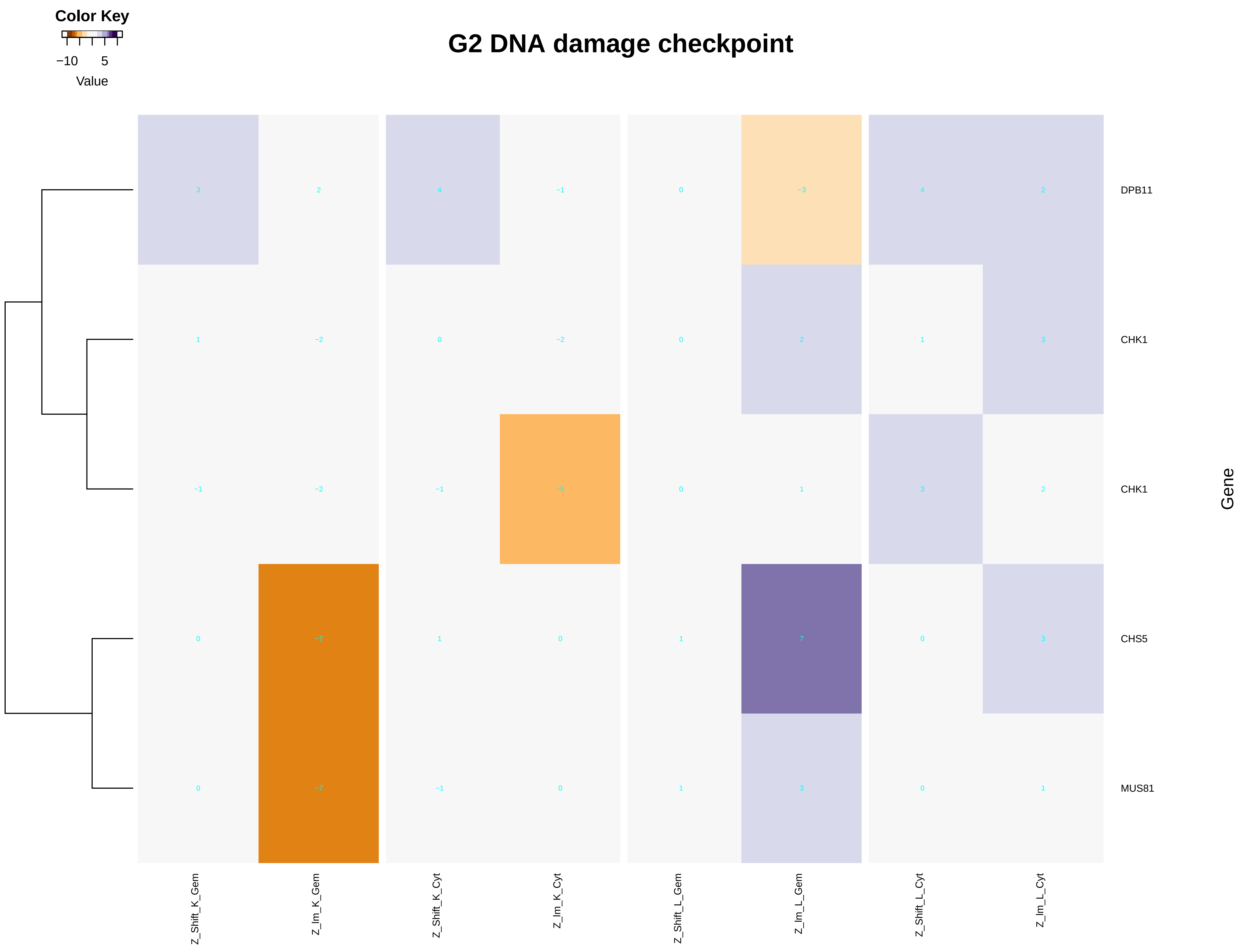
Color Key
−10
5
Value
G2 DNA damage checkpoint
DPB11
CHK1
CHK1
CHS5
MUS81
Gene
Z_Shift_K_Gem
Z_Im_K_Gem
Z_Shift_K_Cyt
Z_Im_K_Cyt
Z_Shift_L_Gem
Z_Im_L_Gem
Z_Shift_L_Cyt
Z_Im_L_Cyt

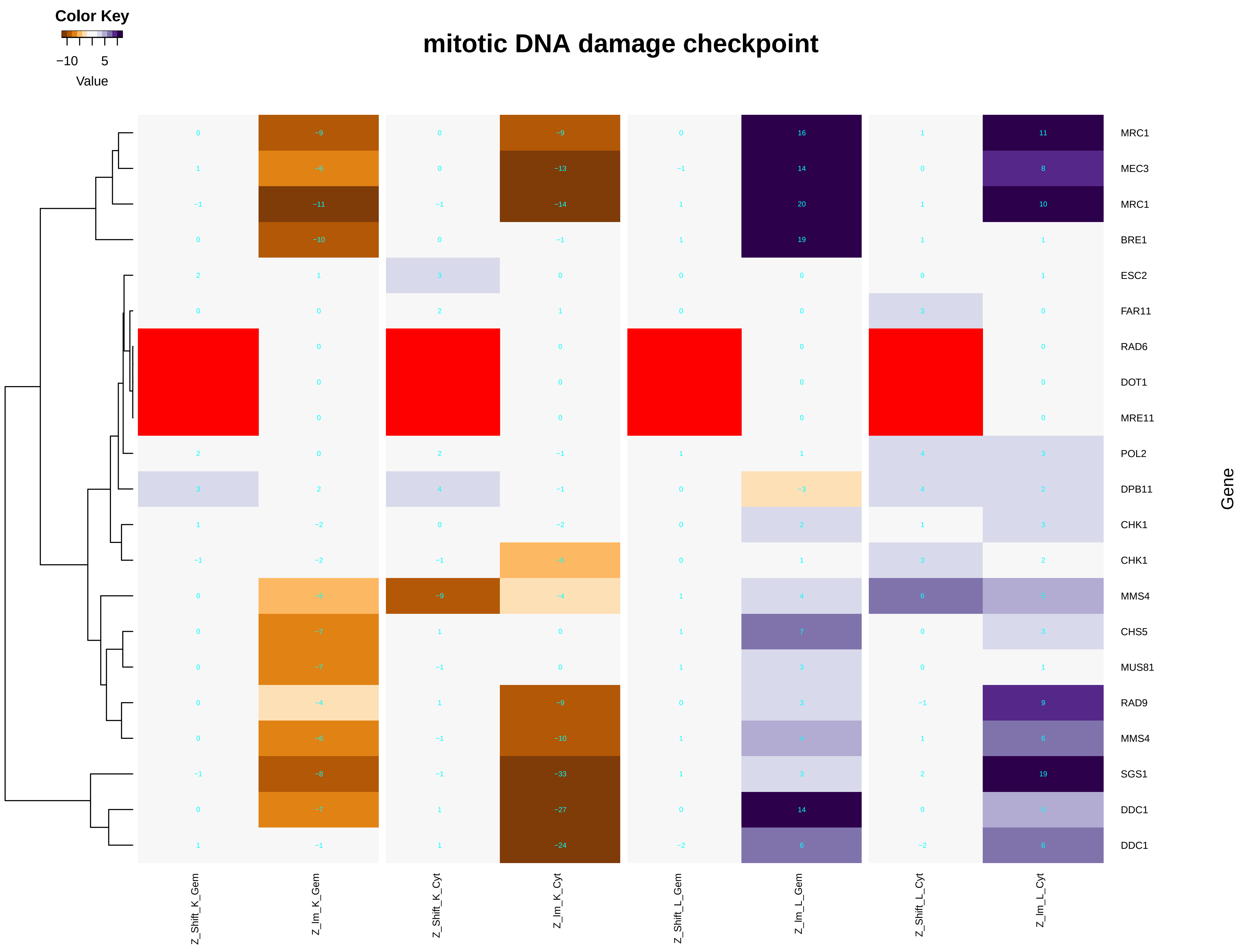

mitotic DNA damage checkpoint
Color Key
−10
5
Value
MRC1
MEC3
MRC1
BRE1
ESC2
FAR11
RAD6
DOT1
MRE11
POL2
DPB11
CHK1
CHK1
MMS4
CHS5
MUS81
RAD9
MMS4
SGS1
DDC1
DDC1
Gene
Z_Shift_K_Gem
Z_Im_K_Gem
Z_Shift_K_Cyt
Z_Im_K_Cyt
Z_Shift_L_Gem
Z_Im_L_Gem
Z_Shift_L_Cyt
Z_Im_L_Cyt

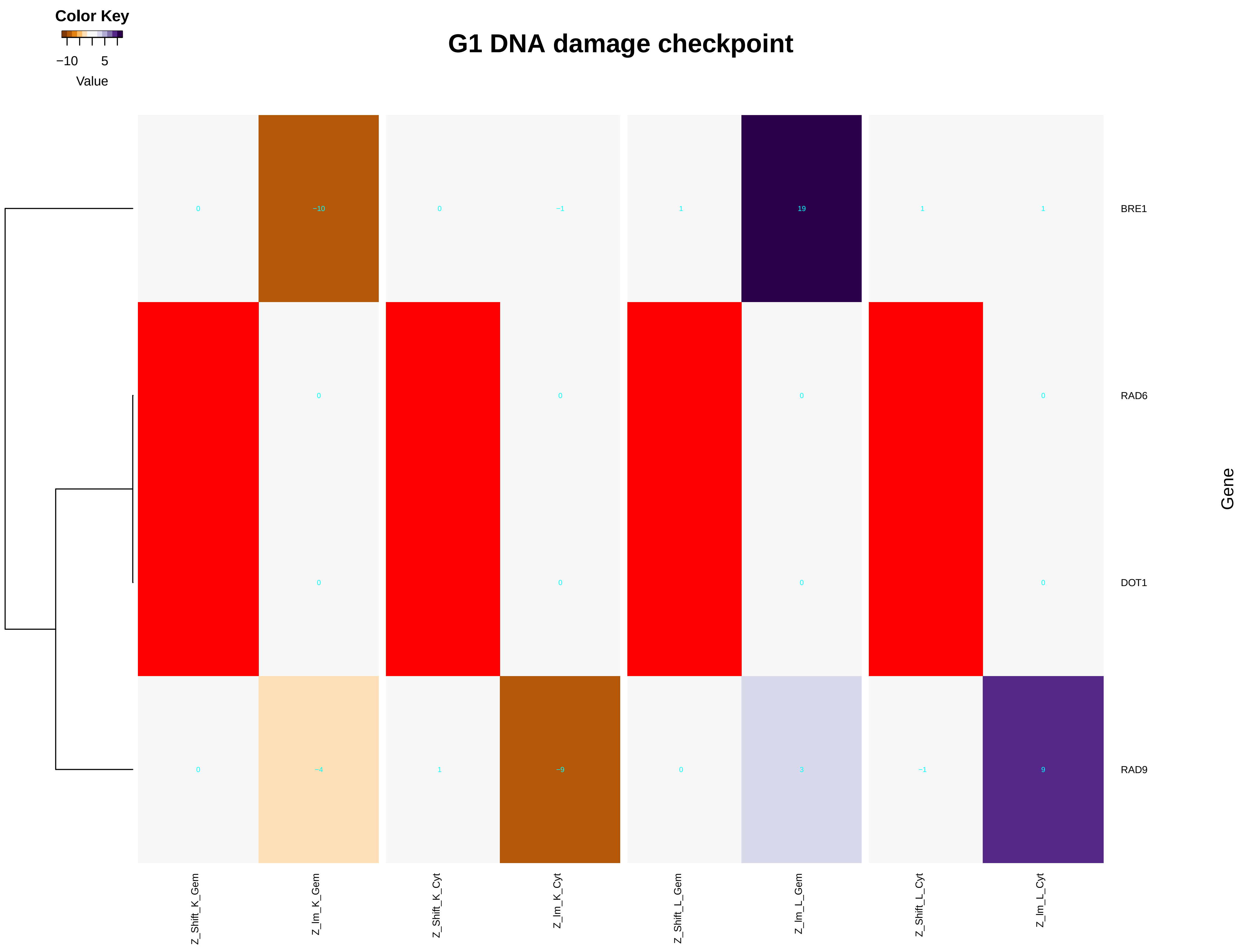
Color Key
−10
5
Value
G1 DNA damage checkpoint
0
−10
0
−1
1
19
1
1
BRE1
0
0
0
0
RAD6
0
0
0
0
DOT1
0
−4
1
−9
0
3
−1
9
RAD9
Gene
Z_Shift_K_Gem
Z_Im_K_Gem
Z_Shift_K_Cyt
Z_Im_K_Cyt
Z_Shift_L_Gem
Z_Im_L_Gem
Z_Shift_L_Cyt
Z_Im_L_Cyt

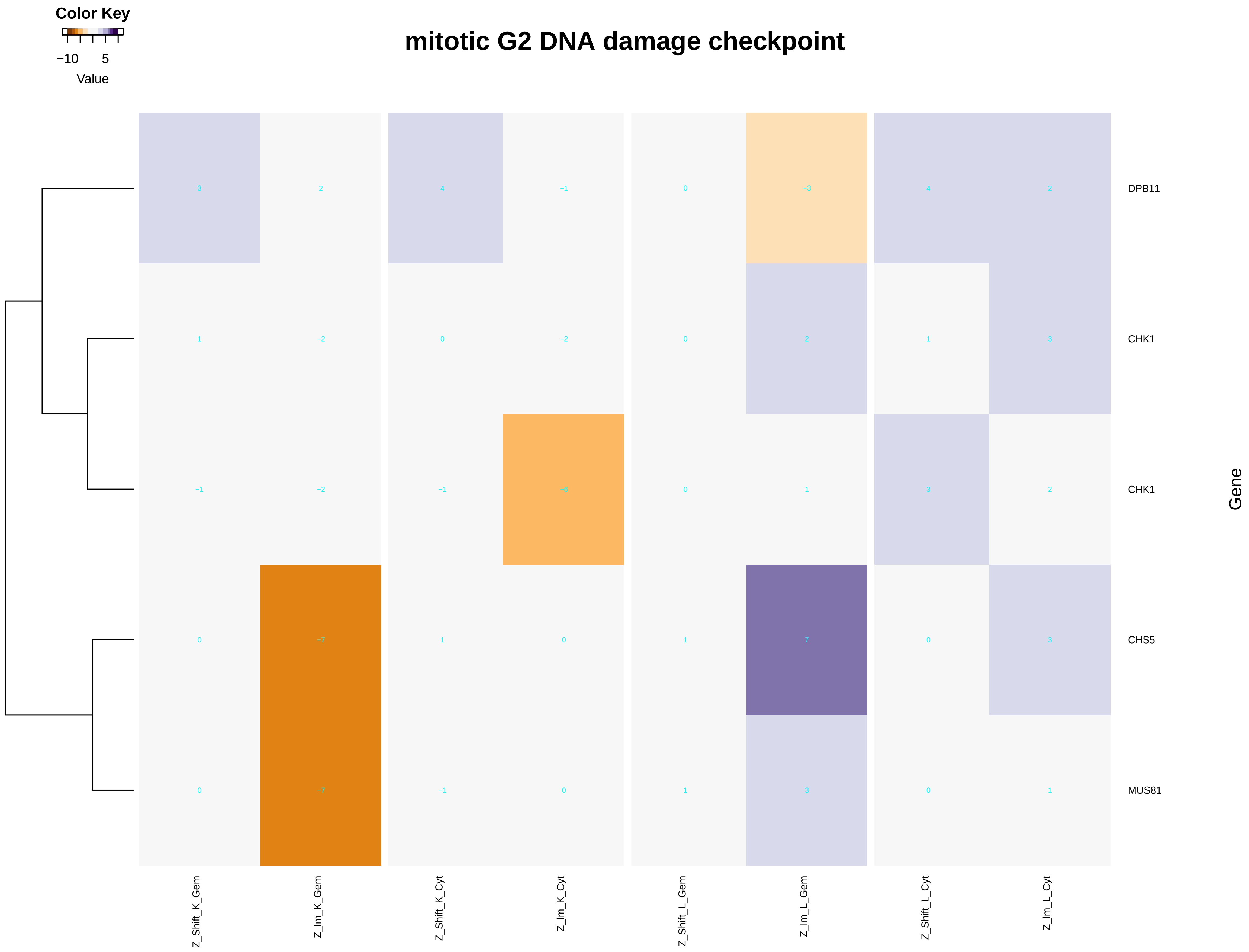

Color Key
−10
5
Value
mitotic G2 DNA damage checkpoint
3 2 4 −1 0 −3 4 2
1 −2 0 −2 0 2 1 3
−1 −2 −1 −6 0 1 3 2
0 −7 1 0 1 7 0 3
0 −7 −1 0 1 3 0 1
DPB11
CHK1
CHK1
CHS5
MUS81
Gene
Z_Shift_K_Gem
Z_Im_K_Gem
Z_Shift_K_Cyt
Z_Im_K_Cyt
Z_Shift_L_Gem
Z_Im_L_Gem
Z_Shift_L_Cyt
Z_Im_L_Cyt

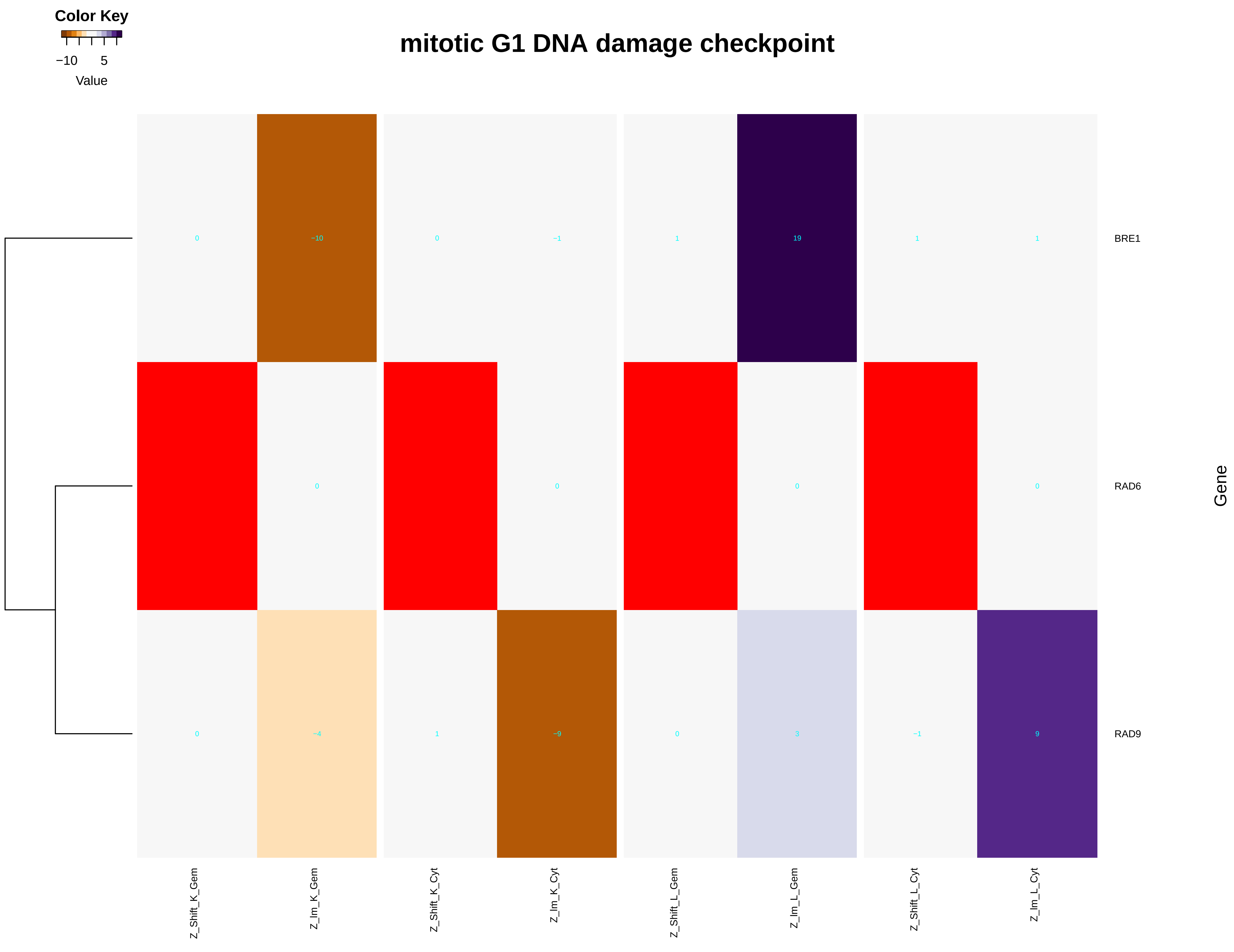

Color Key
−10
5
Value
mitotic G1 DNA damage checkpoint
0
−10
0
−1
1
19
1
1
BRE1
0
0
0
0
RAD6
0
−4
1
−9
0
3
−1
9
RAD9
Gene
Z_Shift_K_Gem
Z_Im_K_Gem
Z_Shift_K_Cyt
Z_Im_K_Cyt
Z_Shift_L_Gem
Z_Im_L_Gem
Z_Shift_L_Cyt
Z_Im_L_Cyt

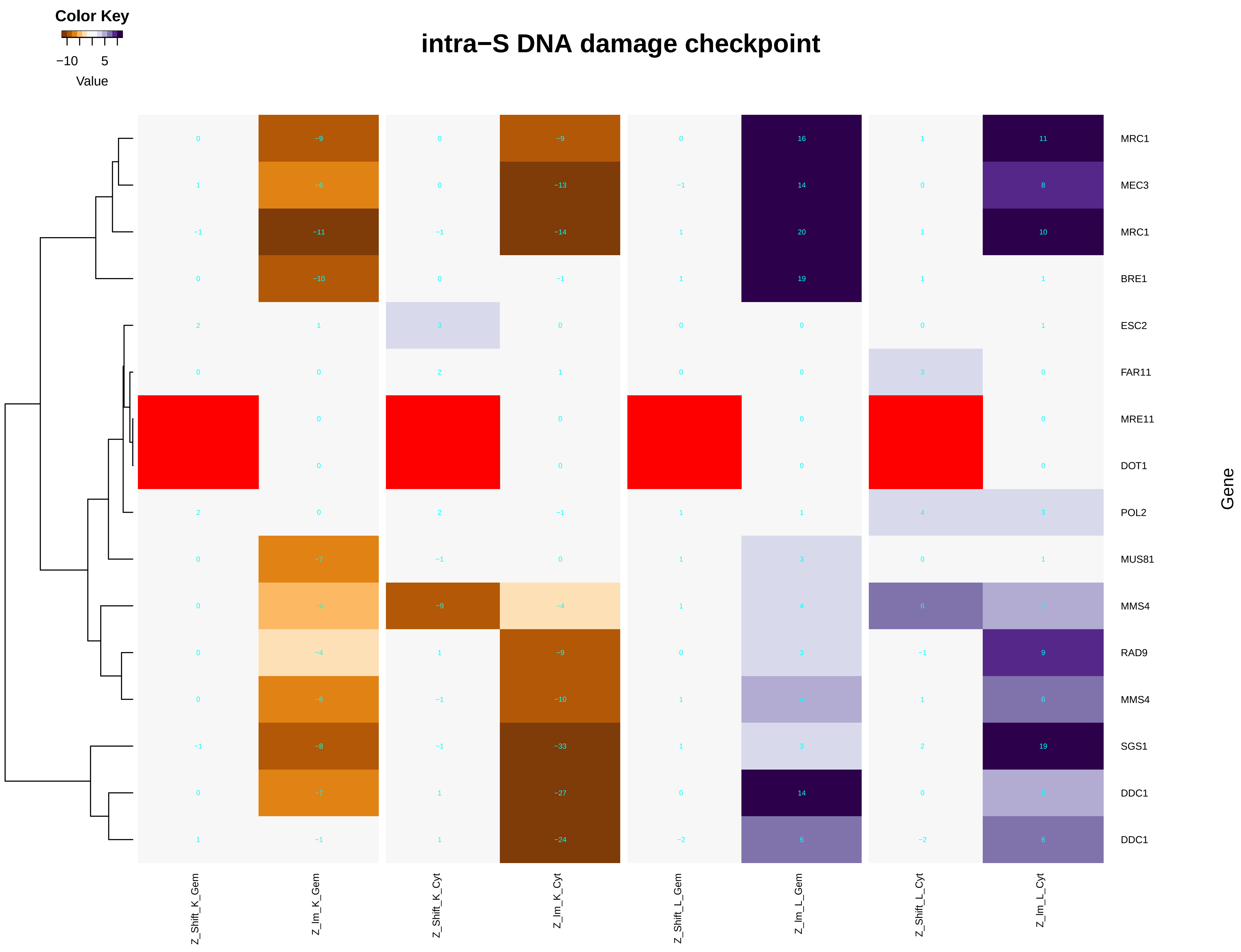

Color Key
−10
5
Value
intra−S DNA damage checkpoint
MRC1
MEC3
MRC1
BRE1
ESC2
FAR11
MRE11
DOT1
POL2
MUS81
MMS4
RAD9
MMS4
SGS1
DDC1
DDC1
Gene
Z_Shift_K_Gem
Z_Im_K_Gem
Z_Shift_K_Cyt
Z_Im_K_Cyt
Z_Shift_L_Gem
Z_Im_L_Gem
Z_Shift_L_Cyt
Z_Im_L_Cyt
